# Persistent ENT Manifestations in Individuals who Recovered from COVID-19: A Systematic Review

**DOI:** 10.1055/s-0043-1777805

**Published:** 2024-03-15

**Authors:** Akriti Sharma, Rohit Kumar Jakhar, Vikas Kakkar, Garima Singal

**Affiliations:** 1 Department of Ear, Nose, and Throat, SGT Medical College, Hospital, and Research Institute, Gurgaon, Haryana, India

**Keywords:** long COVID, SARS-CoV-2, otorhinolaryngological manifestations

## Abstract

**Introduction** Long coronavirus disease (COVID) refers to the persistence of symptoms long after the recovery from the acute phase of the illness, and it is due to the interplay of various inflammatory mechanisms. This has led to emergence of new deficits, including otorhinolaryngological symptoms, in patients wo have recovered from COVID. The plethora of otorhinolaryngological symptoms associated with long COVID are tinnitus, sensorineural hearing loss (SNHL), vertigo, nasal congestion, sinonasal discomfort, hyposmia/anosmia, dysgeusia, sore throat, dry cough, dyspnea, dysphagia, and hoarseness of voice.

**Objective** To evaluate the possible ENT symptoms in patients wo have recovered from COVID and to combine those findings with our experience.

**Data Synthesis** We conducted a search on the PubMed, ENT Cochrane, Web of Science, and Google Scholar databases, and a total of 44 studies were selected for the present review.

**Conclusion** Otorhinolaryngological complications such as tinnitus, SNHL, vertigo, nasal congestion, sinonasal discomfort, hyposmia/anosmia, dysgeusia, sore throat, dry cough, dyspnea, dysphagia, and hoarseness of voice have been widely reported among in long-COVID patients.

## Introduction

In December 2019, the severe acute respiratory syndrome coronavirus 2 (SARS-CoV-2) originated in Wuhan, China. 1 On March 11, 2020, due to its unprecedented global spread, the World Health Organization (WHO) labelled coronavirus disease 2019 (COVID-19), the infection caused by SARS-CoV-2, a pandemic. 2 It is a very aggressive and contagious illness that spreads mostly by aerosols and respiratory droplets. 3 The primary entry points for the virus are the nasal and oral cavities, with the nasal cavity and nasopharynx being the most common sites for viral replication. The virus binds to the angiotensin-converting enzyme 2 (ACE2) receptor and initiates an inflammatory cascade with multisystem involvement. 4 5 6 The symptoms may range from minor ailments to life-threatening complications. In addition to symptoms of the lower respiratory tract, such as cough, fever, and dyspnea, extrapulmonary symptoms affecting the ear, nose, and throat are also often observed. 7

It has been observed that most SARS-CoV-2-positive patients did not require any hospitalization and presented with mild or no symptoms. Among these mild symptoms, alterations in the senses of smell and taste have been observed to occur early. 8

While some of the symptoms diminish with time, others have catastrophic long-term effects that persist even after recovery from the acute infection, resulting in death or significant impairment of functions. “Long COVID” or “post-COVID syndrome” refers to this unusual and unexpected persistence of symptoms long after recovery from the initial illness. 9 Long COVID is associated with a plethora of otorhinolaryngological symptoms, including tinnitus, sensorineural hearing loss (SNHL), vertigo, nasal congestion, sinonasal discomfort, hyposmia/anosmia, dysgeusia, sore throat, dry cough, dyspnea, dysphagia, and hoarseness of voice.

## Methods

We conducted a search on the PubMed, ENT Cochrane, Web of Science, and Google Scholar databases without restrictions regarding the time of publication of the articles, with an emphasis on the most recent reports, using the terms *COVID-19* , *long COVID* , and *otorhinolaryngological manifestation* or *ENT symptoms* . Boolean operators (AND, NOT, and OR) were used to restrict and extend the search. Long COVID can refer to certain symptoms that seem to persist (from three to nine months or longer) in an individual who has recovered from COVID-19. 10 However, some authors 11 also believe these symptoms can persist for years.

Initially, 462 articles were retrieved based on our search parameters. Out of these, 392 publications were excluded, as they were not found to be relevant to the topic in question. Consequently, a total of 70 studies were selected for the present review. The purpose of the current article is to compile the broad spectrum of ear, nose, and throat (ENT) presentations of long COVID and to highlight the diagnosis based on the available literature combined with our experience.

## Review of the Literature

### Otological Symptoms after COVID-19

We analyzed 25 articles in detail to highlight the symptoms and treatment of the otological symptoms of long COVID exclusively. Combined, the data from these studies revealed that out of 1,000 patients who have recovered from COVID, 80 (8%) presented with SNHL, 140 (14%), with tinnitus, 142 (14.2%), with vertigo, 2 (0.2%), with facial paralysis, 19 (1.9%), with an earache, and 58 (5.81%) presented with aural fullness. The broad spectrum of ear symptoms after recovery from COVID are hearing loss, tinnitus, vertigo, facial palsy, earache, and aural fullness. 12 13 14 15 16 17 18 19 20 21 22 23 24 25 26 27 28 29 30 31 32 33 34 35 36

At our center, approximately 22 patients reported to us with symptoms of hearing loss, tinnitus and aural fullness; 4 out of them presented with tinnitus along with hearing loss, 14, with hearing loss without any other symptoms, and 4, with aural fullness. These patients are still being treated and are under follow-up.

### Nasal and Associated Chemosensory Deficit Symptoms Following COVID-19

We analyzed 11 studies in detail to conclude that the most common nasal and associated chemosensory deficit symptoms are smell and taste dysfunctions, such as phantosmia, parosmia, hyposmia, anosmia, phantogeusia, parageusia, ageusia, or rhinitis symptoms, such as nasal discharge, nasal blockage, and sinonasal pain. 33 34 36 37 38 39 40 41 42 43

In each study, the authors observed chemosensory deficit symptoms, including dysfunctions in the senses of smell and taste in 861 patients (31.22%) out of a total of 2,757 patients who have recovered from COVID. Six 33 34 37 40 41 43 out of 10 studies identified rhinitis symptoms in 385 (19.63%) of a total 1,961 patients.

In 2022, Bianco et al. 44 conducted a prospective study on 76 patients for an objective assessment of olfactory function, which revealed that 48 (63.16%) patients had recovered completely, 26 (34.21%) were hyposmic, and 2 (2.63%) were anosmic. The authors reported 44 that the duration of the SARS-CoV-2 infection was observed to be more severe among individuals who did not regain their sense of smell. Hence, they could safely conclude that the duration of the infection negatively correlates with the recovery of olfactory function.

A total of four patients presented to the ENT Outpatient Department (OPD) at our center with nasal complications of long COVID. While 3 out of 4 patients reported with hyposmia, 1 presented with complaints of anosmia. These patients are still under follow up.

### Throat and Laryngeal Symptoms after COVID-19

A total of 10 studies were taken into consideration in order to ascertain that the most prevalent throat and laryngeal symptoms include hoarseness of voice, sore throat, dry throat, trouble swallowing, dyspnea, and cough. 34 36 39 43 45 46 47 48 49

Out of the 1,826 subjects who have recovered from COVID included in these studies, symptoms of hoarseness of voice were observed in 110 patients (6.02%), sore throat, in 214 (11.72%), dry throat, in 26 (1.42%), difficulty in swallowing in 33 (1.81%), dyspnea, in 313 (17.14%), and cough, in 344 patients (18.84%).

In 4 out of 10 studies, 45 46 48 49 the authors also reported history of intubation, accounting for 52 of 207 (25.12%) patients who had recovered from COVID.

At our center, we have only observed one patient with a finding of vocal cord paresis. We have not observed any other laryngeal complications of long COVID.

## Discussion

The infection by SARS-CoV-2 (COVID-19) is a highly contagious illness with catastrophic effects, which has caused great mortality and morbidity on a global scale. About 80% of the afflicted patients presented with mild to moderate disease, whereas 5% of the cases were considered critical. 50 Long COVID or post-COVID refers to the persistence of many symptoms weeks or months after the infection by SARS-CoV-2, regardless of the viral state. 51 Long-term COVID may be ongoing or have a relapsing, remitting nature. 52 There may be a continuation of one or more acute COVID symptoms, or the emergence of new symptoms. Most individuals with post-COVID syndrome are polymerase chain reaction (PCR) negative, which indicates recovery of the virus. The lag between microbiological and clinical recovery is referred to as post-COVID syndrome. 53 Most long-term COVID patients recover biochemically and radiologically. 54

Long COVID may be caused by the persistence of inflammatory process or immune response/autoantibody production, the persistence of the virus in the body, the sequelae of organ dysfunction, and the varying time involved for the regeneration of each organ, postintensive care syndrome, complications due to the COVID infection or complications due to underlying diseases or adverse effects of the medications used. Therefore, multiple pathways contribute to the development of long COVID in various patients. 55 56 Long COVID patients commonly experience extreme fatigue, shortness of breath, cough, chest discomfort, palpitations, headache, joint pain, muscle ache and fatigue, insomnia, diarrhea, poor balance and gait, neurocognitive disorders such as memory and attention problems, and a decline in quality of life. 57

Tinnitus, SNHL, vertigo, facial paralysis, earache, aural fullness, gustatory and olfactory dysfunctions, rhinitis symptoms such as nasal discharge and nasal obstruction, dyspnea, cough, hoarseness of voice, dysphagia, sore throat, and dry throat are otorhinolaryngological symptoms of long COVID. based on these symptoms, neuro-otorhinolaryngological symptoms can be explained through various mechanisms. This might be due to hypoxia caused by respiratory failure, immune-mediated injury, coagulation problems, or direct viral invasion. 58

The SARS-Cov-2 virus can also cause direct damage by binding to the ACE2 receptor, which is commonly expressed in the nasal, laryngeal, or tracheal tracts, as well as in the neuro-olfactory epithelium, the lungs, the Eustachian tube, the middle ear, the cochlear hair cells, the oral cavity, including the taste buds, the endothelium of blood vessels, and the thyroid gland. This virus may directly infiltrate tissue and, since it is neuroinvasive and neurotrophic, it can cause axonal transport, thus causing long-term damage to individual neurons. Involvement of the nasal, laryngeal, and tracheal tracts, as well as the neuro-olfactory epithelium, results in rhinitis, anosmia, sore throat, dry throat, dysphagia, hoarseness of voice, and cough. Involvement of the lungs results in dyspnea, hypoxic symptoms, and hemoptysis.

Involvement of the Eustachian tube and middle ear causes earache and aural fullness. The combination of cochlear hair cells with the involvement of the vestibulocochlear nerve and facial nerve results in cochlear neuritis/labrynthitis or vestibular neuritis, hence further adding to the symptoms of SNHL, tinnitus, vertigo, and facial nerve paralysis. Involvement of the oral cavity, including the taste buds, results in gustatory impairment. Due to the predilection of the virus for the thyroid gland through the ACE 2 receptors, thyroid involvement results in subacute thyroiditis. 59 60 61 62

Involvement of terminal cochlear artery supply resulting in SNHL and tinnitus, as a result of indirect ischemia injury, is another possible cause of audiovestibular symptoms. There is degradation of the basal lamina of endothelial cells, congestion of blood vessels, and an infiltration of macrophages, astrocytes, and lymphocytes around the blood vessels. This also causes microhemorrhages in the parenchyma and labyrinth of the brain. 17 19 63 There is an aberrant immune response characterized by the formation of a significant number of autoantibodies directed against blood vessels, which may lead to autoimmune thrombocytopenia and the subsequent development of labyrinthine ischemia due to hypoxia. 64 65

In the etiopathogenesis of neuro-otorhinolaryngological symptoms, inflammatory cytokines and their overproduction also play a role. Their excessive production is the cause of chronic symptoms. Interleukin 6 generated by the lymphocytes, the fibroblasts, and the bronchial epithelium during SARS-CoV-2 infection may control synaptic plasticity, alter cerebral blood flow, and induce neuroinflammation. 66 67

In addition to the aforementioned processes, the use of ototoxic medicines such as hydroxychloroquine, favipiravir, azithromycin, and furosemide to treat COVID might result in reversible or irreversible hearing impairment. 68 69 70 71

In our opinion, the treatment for the otorhinolaryngological symptoms of long-term COVID requires a multidisciplinary strategy that includes examination, symptomatic therapy, treatment of underlying issues, and long-term monitoring of ongoing symptoms for the identification and timely intervention regarding the complications.

Monitored steroids at a low dose can be used for the treatment of the inflammatory processes such as neuroinflammation. However, steroids must be administered with caution and after taking complete medical history of the patient in detail. The uncontrolled and injudicious use of steroids is has also been observed to result in sequelae like mucormycosis in long-COVID patients.

## Conclusion

Numerous complications of long COVID have been reported in the literature, with ENT complications contributing greatly to the impact on the lives of patients after the pandemic. We have observed that otorhinolaryngological complications, such as tinnitus, SNHL, vertigo, nasal congestion, sinonasal discomfort, hyposmia/anosmia, dysgeusia, sore throat, dry cough, dyspnea, dysphagia, hoarseness of voice, and hypothyroidism have been widely reported. We believe that although “wait and watch” is the immediate answer to any mild complaint, steroids and nootropic medicines might play some role in the treatment of certain complications mentioned in the present review. However, a detailed medical history and adequate dosage and duration of medication use are the mainstay of the management of most ENT complications of long COVID.

## References

[1] El-Anwar M W Elzayat S Fouad Y A ENT manifestation in COVID-19 patients Auris Nasus Larynx 2020 47 04 559 564 32586739 10.1016/j.anl.2020.06.003 PMC7294298

[2] China Medical Treatment Expert Group for Covid-19 Guan W J Ni Z Y Hu Y Clinical characteristics of coronavirus disease 2019 in China N Engl J Med 2020 382 18 1708 1720 32109013 10.1056/NEJMoa2002032 PMC7092819

[3] Heller L Mota C R Greco D B COVID-19 faecal-oral transmission: Are we asking the right questions? Sci Total Environ 2020 729 138919 32353720 10.1016/j.scitotenv.2020.138919 PMC7182518

[4] Gengler I Wang J C Speth M M Sedaghat A R Sinonasal pathophysiology of SARS-CoV-2 and COVID-19: A systematic review of the current evidence Laryngoscope Investig Otolaryngol 2020 5 03 354 359 10.1002/lio2.384 PMC7262250 32587887

[5] Hoffmann M Kleine-Weber H Schroeder S SARS-CoV-2 Cell entry depends on ACE2 and TMPRSS2 and is blocked by a clinically proven protease inhibitor Cell 2020 181 02 271 2.8E10 32142651 10.1016/j.cell.2020.02.052 PMC7102627

[6] Hussman J P Cellular and molecular pathways of COVID-19 and potential points of therapeutic intervention Front Pharmacol 2020 11 1169 32848776 10.3389/fphar.2020.01169 PMC7406916

[7] Tuang G J Abdul Wahab A F Husain S Otolaryngology manifestations of COVID-19: a contemporary viewpoint Postgrad Med J 2022 98(e2):e97 e98 37066528 10.1136/postgradmedj-2021-140169

[8] Bianco M R Modica D M Drago G D Alteration of smell and taste in asymptomatic and symptomatic COVID-19 patients in sicily, italy Ear Nose Throat J 2021 100(2_suppl, suppl)182S 185S 33350319 10.1177/0145561320981447

[9] National Institute for Health and Care Excellence COVID-19 rapid guideline: managing the long-term effects of COVID-19 NICE guideline; c2020 https://www.nice.org.uk/guidance/ng188 33555768

[10] https://www.who.int/podcasts/series/science-in-5/episode–47−post-covid-19-condition

[11] Davis H E McCorkell L Vogel J M Topol E J Long COVID: major findings, mechanisms and recommendations Nat Rev Microbiol 2023 21 03 133 146 36639608 10.1038/s41579-022-00846-2 PMC9839201

[12] Kilic O Kalcioglu M T Cag Y Could sudden sensorineural hearing loss be the sole manifestation of COVID-19? An investigation into SARS-COV-2 in the etiology of sudden sensorineural hearing loss Int J Infect Dis 2020 97 208 211 32535294 10.1016/j.ijid.2020.06.023 PMC7289736

[13] Degen C Lenarz T Willenborg K Acute Profound Sensorineural Hearing Loss After COVID-19 Pneumonia Mayo Clin Proc 2020 95 08 1801 1803 32753155 10.1016/j.mayocp.2020.05.034 PMC7275185

[14] Abdel Rhman S Abdel Wahid A COVID -19 and sudden sensorineural hearing loss, a case report Otolaryngol Case Rep 2020 16 100198 34957357 10.1016/j.xocr.2020.100198 PMC7342036

[15] Lang B Hintze J Conlon B Coronavirus disease 2019 and sudden sensorineural hearing loss J Laryngol Otol 2020 134 1 3 10.1017/S0022215120002145 PMC7684203 33000716

[16] Koumpa F S Forde C T Manjaly J G Sudden irreversible hearing loss post COVID-19 BMJ Case Rep 2020 13 11 e238419 10.1136/bcr-2020-238419 PMC7554505 33051251

[17] Lamounier P Franco Gonçalves V Ramos H VL A 67-Year-Old Woman with Sudden Hearing Loss Associated with SARS-CoV-2 Infection Am J Case Rep 2020 21 e927519 33139689 10.12659/AJCR.927519 PMC7650213

[18] Karimi-Galougahi M Naeini A S Raad N Mikaniki N Ghorbani J Vertigo and hearing loss during the COVID-19 pandemic - is there an association? Acta Otorhinolaryngol Ital 2020 40 06 463 465 32519994 10.14639/0392-100X-N0820 PMC7889249

[19] Chern A Famuyide A O Moonis G Lalwani A K Bilateral Sudden Sensorineural Hearing Loss and Intralabyrinthine Hemorrhage in a Patient With COVID-19 Otol Neurotol 2021 42 01 e10 e14 33301283 10.1097/MAO.0000000000002860 PMC7737860

[20] Aasfara J Hajjij A Bensouda H Ouhabi H Benariba F A unique association of bifacial weakness, paresthesia and vestibulocochlear neuritis as post-COVID-19 manifestation in pregnant women: a case report Pan Afr Med J 2021 38 30 33777298 10.11604/pamj.2021.38.30.27646 PMC7955605

[21] Beckers E Chouvel P Cassetto V Mustin V Sudden sensorineural hearing loss in COVID-19: A case report and literature review Clin Case Rep 2021 9 04 2300 2304 33936683 10.1002/ccr3.4019 PMC8077296

[22] Edwards M Muzaffar J Naik P Coulson C Catastrophic bilateral sudden sensorineural hearing loss following COVID-19 BMJ Case Rep 2021 14 06 e243157 10.1136/bcr-2021-243157 PMC8230962 34167987

[23] Ozer F Alkan O Simultaneous Sudden Hearing Loss and Peripheral Facial Paralysis in a Patient with COVID-19Ear Nose Throat 2021 10.1177/01455613211028094 34219500

[24] Ricciardiello F Pisani D Viola P Sudden Sensorineural Hearing Loss in Mild COVID-19: Case Series and Analysis of the Literature Audiology Res 2021 11 03 313 326 10.3390/audiolres11030029 PMC8293051 34287226

[25] Gerstacker K Speck I Riemann S Aschendorff A Knopf A Arndt S Deafness after COVID-19? HNO 2021 69 02 92 95 34019138 10.1007/s00106-021-01041-0 PMC8138955

[26] Jeong M Ocwieja K E Han D Direct SARS-CoV-2 infection of the human inner ear may underlie COVID-19-associated audiovestibular dysfunction Commun Med (Lond) 2021 1 01 44 34870285 10.1038/s43856-021-00044-w PMC8633908

[27] Pokharel S Tamang S Pokharel S Mahaseth R K Sudden sensorineural hearing loss in a post-COVID-19 patient Clin Case Rep 2021 9 10 e04956 34703602 10.1002/ccr3.4956 PMC8520694

[28] Asfour L Kay-Rivest E Roland J T Jr Cochlear implantation for single-sided deafness after COVID-19 hospitalization Cochlear Implants Int 2021 22 06 353 357 34151741 10.1080/14670100.2021.1936364

[29] Raymaekers V D'hulst S Herijgers D Susac syndrome complicating a SARS-CoV-2 infection J Neurovirol 2021 27 06 954 959 34735693 10.1007/s13365-021-01022-7 PMC8567974

[30] Viola P Ralli M Pisani D Tinnitus and equilibrium disorders in COVID-19 patients: preliminary results Eur Arch Otorhinolaryngol 2021 278 10 3725 3730 33095432 10.1007/s00405-020-06440-7 PMC7582442

[31] Bhatta S Sharma S Sharma D Study of hearing status in COVID-19 patients: a multicentered review Indian J Otolaryngol Head Neck Surg 2021 1 7 34277385 10.1007/s12070-021-02710-w PMC8274964

[32] Gallus R Melis A Rizzo D Audiovestibular symptoms and sequelae in COVID-19 patients J Vestib Res 2021 31 05 381 387 33579886 10.3233/VES-201505

[33] Kökoğlu K Tektaş N Baktir-Okcesiz F EM Şahin Mİ Mild and moderate COVID-19 disease does not affect hearing function permanently: a cross-sectional study ınvolving young and middle-aged healthcare givers Eur Arch Otorhinolaryngol 2021 278 09 3299 3305 34185143 10.1007/s00405-021-06883-6 PMC8238665

[34] Thrane J F Britze A Fjaeldstad A W Incidence and duration of self-reported hearing loss and tinnitus in a cohort of COVID-19 patients with sudden chemosensory loss: a STROBE observational studyEur Ann Otorhinolaryngol Head Neck Dis 2021:S1879-7296(21)00224-6 10.1016/j.anorl.2021.07.012 PMC8482223 34602376

[35] Gedik Ö Hüsam H Başöz M Tas N Aksoy F The effect of coronavirus disease 2019 on the hearing system J Laryngol Otol 2021 135 09 810 814 34344488 10.1017/S0022215121001961

[36] Graham E L Clark J R Orban Z S Persistent neurologic symptoms and cognitive dysfunction in non-hospitalized Covid-19 “long haulers” Ann Clin Transl Neurol 2021 8 05 1073 1085 33755344 10.1002/acn3.51350 PMC8108421

[37] Boscolo-Rizzo P Guida F Polesel J Sequelae in adults at 12 months after mild-to-moderate coronavirus disease 2019 (COVID-19) Int Forum Allergy Rhinol 2021 11 12 1685 1688 34109765 10.1002/alr.22832 PMC9291310

[38] Havervall S Rosell A Phillipson M Symptoms and Functional Impairment Assessed 8 Months After Mild COVID-19 Among Health Care Workers JAMA 2021 325 19 2015 2016 33825846 10.1001/jama.2021.5612 PMC8027932

[39] Augustin M Schommers P Stecher M Post-COVID syndrome in non-hospitalised patients with COVID-19: a longitudinal prospective cohort study Lancet Reg Health Eur 2021 6 100122 34027514 10.1016/j.lanepe.2021.100122 PMC8129613

[40] Vaira L A Gessa C Deiana G The Effects of Persistent Olfactory and Gustatory Dysfunctions on Quality of Life in Long-COVID-19 Patients Life (Basel) 2022 12 02 141 35207429 10.3390/life12020141 PMC8878431

[41] Carvalho-Schneider C Laurent E Lemaignen A Follow-up of adults with noncritical COVID-19 two months after symptom onset Clin Microbiol Infect 2021 27 02 258 263 33031948 10.1016/j.cmi.2020.09.052 PMC7534895

[42] Ercoli T Masala C Pinna I Qualitative smell/taste disorders as sequelae of acute COVID-19 Neurol Sci 2021 42 12 4921 4926 34557966 10.1007/s10072-021-05611-6 PMC8459812

[43] Lackermair K Wilhelm K William F The prevalence of persistent symptoms after COVID-19 disease Dtsch Arztebl Int 2022 119 10 175 176 35583039 10.3238/arztebl.m2022.0125 PMC9215271

[44] Bianco M R Ralli M Minni A Greco A de Vincentiis M Allegra E Evaluation of olfactory dysfunction persistence after COVID-19: a prospective study Eur Rev Med Pharmacol Sci 2022 26 03 1042 1048 35179771 10.26355/eurrev_202202_28014

[45] Halpin S J McIvor C Whyatt G Postdischarge symptoms and rehabilitation needs in survivors of COVID-19 infection: A cross-sectional evaluation J Med Virol 2021 93 02 1013 1022 32729939 10.1002/jmv.26368

[46] El Kik A Eid H Aoun Bacha Z Post-COVID-19 paradoxical vocal cord movement and dysfunctional dysphonia: A clinical case Respir Med Case Rep 2022 39 101710 35874177 10.1016/j.rmcr.2022.101710 PMC9287584

[47] Dassie-Leite A P Gueths T P Ribeiro V V Pereira E C Martins P DN Daniel C R Vocal Signs and Symptoms Related to COVID-19 and Risk Factors for their Persistence J Voice 2021:S0892-199721 253-8 34583881 10.1016/j.jvoice.2021.07.013 PMC8354794

[48] Neevel A J Smith J D Morrison R J Hogikyan N D Kupfer R A Stein A P Postacute COVID-19 Laryngeal Injury and Dysfunction OTO Open 2021 5 03 X211041040 10.1177/2473974X211041040 PMC8392819 34458661

[49] Kang Y R Oh J Y Lee J H Small P M Chung K F Song W J Long-COVID severe refractory cough: discussion of a case with 6-week longitudinal cough characterization Asia Pac Allergy 2022 12 02 e19 35571551 10.5415/apallergy.2022.12.e19 PMC9066079

[50] Wu Z McGoogan J M Characteristics of and important lessons from the coronavirus disease 2019 (COVID-19) outbreak in China: summary of a report of72 314 cases from the Chinese Center for Disease Control and Prevention JAMA 2020 323 13 1239 1242 32091533 10.1001/jama.2020.2648

[51] Geddes L Why strange and debilitating coronavirus symptoms can last for monthsNew Sci2020 https://www.newscientist.com/article/mg24632881-400-why-strange-and-debilitatingcoronavirus-symptoms-can-last-for-months/

[52] Nabavi N Long covid: How to define it and how to manage it BMJ 2020 370 m3489 32895219 10.1136/bmj.m3489

[53] Garg P Arora U Kumar A Wig N The “post-COVID” syndrome: how deep is the damage?J Med Virol 2020 10.1002/jmv.26465 PMC7461449 32852801

[54] Greenhalgh T Knight M A'Court C Buxton M Husain L Management of post-acute covid-19 in primary care BMJ 2020 370 m3026 32784198 10.1136/bmj.m3026

[55] Colafrancesco S Alessandri C Conti F Priori R COVID-19 gone bad: A new character in the spectrum of the hyperferritinemic syndrome? Autoimmun Rev 2020 19 07 102573 32387470 10.1016/j.autrev.2020.102573 PMC7199723

[56] Tay M Z Poh C M Rénia L MacAry P A Ng L FP The trinity of COVID-19: immunity, inflammation and intervention Nat Rev Immunol 2020 20 06 363 374 32346093 10.1038/s41577-020-0311-8 PMC7187672

[57] Raveendran A V Jayadevan R Sashidharan S Long COVID: An overview Diabetes Metab Syndr 2021 15 03 869 875 33892403 10.1016/j.dsx.2021.04.007 PMC8056514

[58] Fancello V Hatzopoulos S Corazzi V SARS-CoV-2 (COVID-19) and audio-vestibular disordersInt J Immunopathol Pharmacol 2021;35:20587384211027373 10.1177/20587384211027373 PMC8216371 34142589

[59] De Luca P Di Stadio A Colacurcio V Long COVID, audiovestibular symptoms and persistent chemosensory dysfunction: a systematic review of the current evidence Acta Otorhinolaryngol Ital 2022 42(2, Suppl. 1)S87 S93 35763279 10.14639/0392-100X-suppl.1-42-2022-10 PMC9137376

[60] Fancello V Fancello G Hatzopoulos S Sensorineural Hearing Loss Post-COVID-19 Infection: An Update Audiology Res 2022 12 03 307 315 10.3390/audiolres12030032 PMC9219889 35735365

[61] Safwan M Vijayan K N Jithu T G Post-Coronavirus Disease 2019 De Quervain's Thyroiditis – A Case Report and Literature Review Innovare Journal of Medical Sciences 2021 9 02 4 6

[62] Muller I Cannavaro D Dazzi D SARS-CoV-2-related atypical thyroiditis Lancet Diabetes Endocrinol 2020 8 09 739 741 32738929 10.1016/S2213-8587(20)30266-7 PMC7392564

[63] Lee M-H Perl D P Nair G Microvascular Injury in the Brains of Patients with Covid-19 N Engl J Med 2021 384 05 481 483 33378608 10.1056/NEJMc2033369 PMC7787217

[64] Angileri F Legare S Marino Gammazza A Conway de Macario E Jl Macario A Cappello F Molecular mimicry may explain multi-organ damage in COVID-19 Autoimmun Rev 2020 19 08 102591 32535095 10.1016/j.autrev.2020.102591 PMC7289093

[65] Kaliyappan K Chen Y C Krishnan Muthaiah V P Vestibular Cochlear Manifestations in COVID-19 Cases Front Neurol 2022 13 850337 35370886 10.3389/fneur.2022.850337 PMC8971520

[66] Gruol D L IL-6 regulation of synaptic function in the CNS Neuropharmacology 2015 96(Pt A):42 54 25445486 10.1016/j.neuropharm.2014.10.023 PMC4446251

[67] De Luca P Scarpa A Ralli M Auditory disturbances and SARS-CoV-2 infection: brain inflammation or cochlear affection? Systematic review and discussion of potential pathogenesis Front Neurol 2021 12 707207 34421805 10.3389/fneur.2021.707207 PMC8373381

[68] Johansen P B Gran J T Ototoxicity due to hydroxychloroquine: report of two cases Clin Exp Rheumatol 1998 16 04 472 474 9706431

[69] Cianfrone G Pentangelo D Cianfrone F Pharmacological drugs inducing ototoxicity, vestibular symptoms and tinnitus: a reasoned and updated guide Eur Rev Med Pharmacol Sci 2011 15 06 601 636 21796866

[70] Elfiky A A Ribavirin, Remdesivir, Sofosbuvir, Galidesivir, and Tenofovir against SARS-CoV-2 RNA dependent RNA polymerase (RdRp): A molecular docking study Life Sci 2020 253 117592 32222463 10.1016/j.lfs.2020.117592 PMC7102646

[71] Ciorba A Corazzi V Skarżyński P H Don't forget ototoxicity during the SARS-CoV-2 (Covid-19) pandemic!Int J Immunopathol Pharmacol 2020;34:2058738420941754 10.1177/2058738420941754 PMC7357052 32649262

